# Supplementary material for: Negative selection maintains transcription factor binding motifs in human cancer
Source: BMC Genomics. 2016 Jun 23;17(Suppl 2):395. doi: 10.1186/s12864-016-2728-9 (PMC4928157; doi:10.1186/s12864-016-2728-9)
Supplement: Additional file 3: Figure S1. — Relative frequencies of non-coding mutation contexts in different cancer types. (top panel) Three cancer types with the largest number of mutation calls exhibit different mutation signatures. (bottom panel) Overall comparison of non-coding mutation signatures in 10 cancer types. The samples with lower numbers of total mutation calls display extreme contexts distributions. Cancer types are sorted by the total number of mutation calls. Mutations are grouped by the substitution (X > Y), the 5' and 3' nucleotides are shown in a lexicographical order. (PDF 246 kb) [file 12864_2016_2728_MOESM3_ESM.pdf]

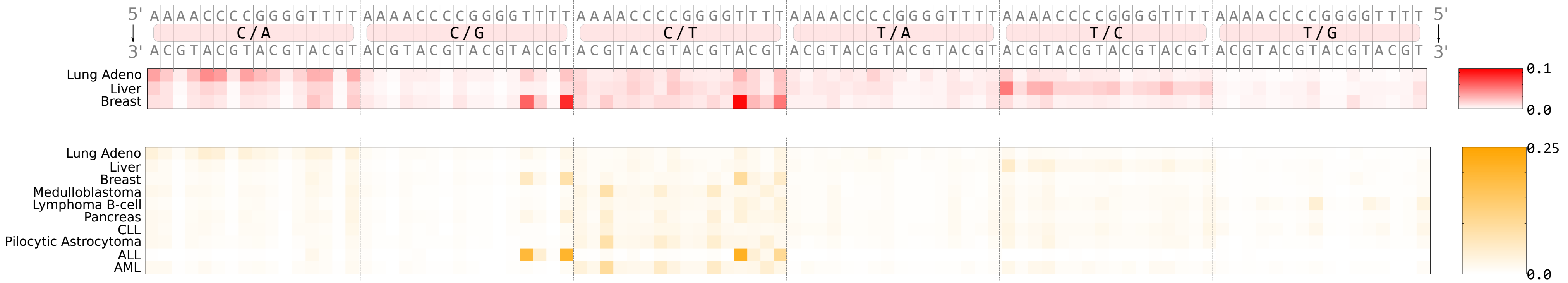

Additional file 3 Figure S1.

Relative frequencies of non-coding mutation contexts in different cancer types.

(top panel) Three cancer types with the largest number of mutation calls exhibit different mutation signatures.
(bottom panel) Overall comparison of non-coding mutation signatures in 10 cancer types.
The samples with lower numbers of total mutation calls display extreme contexts distributions. Cancer types are sorted by the total number of mutation calls. Mutations are grouped by the substitution (X > Y), the 5' and 3' nucleotides are shown in a lexicographical order.
