# Supplementary material for: Negative selection maintains transcription factor binding motifs in human cancer
Source: BMC Genomics. 2016 Jun 23;17(Suppl 2):395. doi: 10.1186/s12864-016-2728-9 (PMC4928157; doi:10.1186/s12864-016-2728-9)
Supplement: Additional file 6: Figure S2. — Agreement between shuffle and genomic control data for detection of conserved motifs (negative selection) and motifs frequently altered (targeted) by mutations (positive selection). X axis shows the number of motifs, stacked bars show the number of significant motifs passing P < 0.05 for shuffle only (green), genomic only (light blue) and both (deep blue) controls. Data for three cancer types are shown. Panels: (A) Affinity loss. (B) Affinity gain. (PDF 72 kb) [file 12864_2016_2728_MOESM6_ESM.pdf]

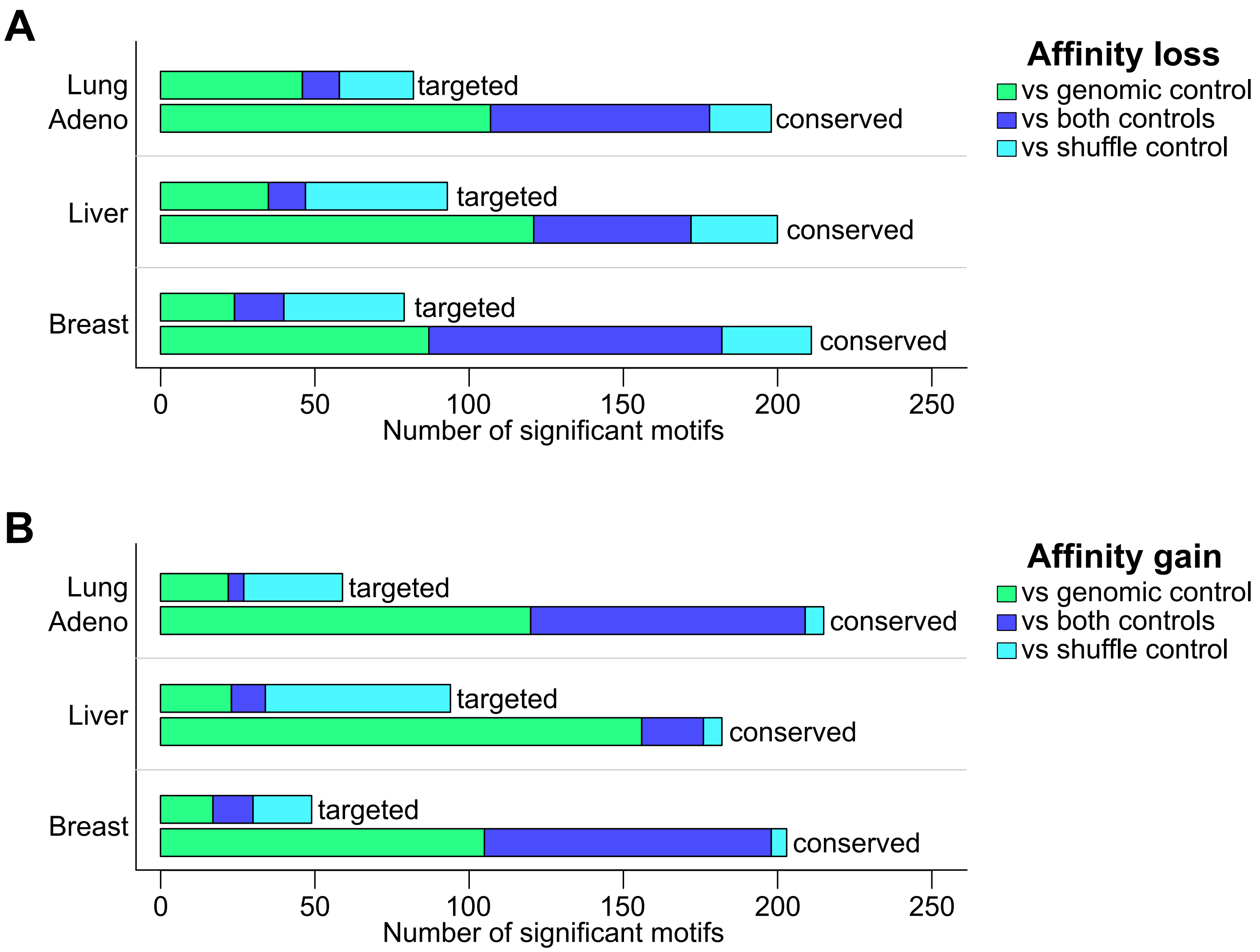


**Additional file 6 Figure S2.**
Agreement between shuffle and genomic control data for detection of conserved motifs (negative selection) and motifs frequently altered (targeted) by mutations (positive selection).

X axis shows the number of motifs, stacked bars show the number of significant motifs passing $P < 0.05$ for shuffle only (green), genomic only (light blue) and both (deep blue) controls. Data for three cancer types are shown.

Panels: (**A**) Affinity loss. (**B**) Affinity gain.
